# The Impact of the Common Elements Treatment Approach on HIV Treatment Outcomes Among Women Experiencing Intimate Partner Violence in South Africa: A Randomized Trial

**DOI:** 10.1002/jia2.70172

**Published:** 2026-07-27

**Authors:** Matthew P. Fox, Jeremy C. Kane, Sithabile Mngadi‐Ncube, Amy Zheng, Kristina Metz, Pertunia Manganye, Lawrence Long, Ross Greener, Srishti Sardana, Taylor Allen, Donald M. Thea, Bridgette Goeieman, Marnie Vujovic, Laura K. Murray, Sophie Pascoe

**Affiliations:** ^1^ Department of Global Health Boston University School of Public Health Boston Massachusetts USA; ^2^ Department of Epidemiology Boston University School of Public Health Boston Massachusetts USA; ^3^ Health Economics and Epidemiology Research Office Department of Internal Medicine School of Clinical Medicine Faculty of Health Sciences University of the Witwatersrand Johannesburg South Africa; ^4^ Department of Epidemiology Columbia University Mailman School of Public Health New York New York USA; ^5^ Brown University Health Providence Rhode Island USA; ^6^ Department of Mental Health Johns Hopkins Bloomberg School of Public Health Baltimore Maryland USA; ^7^ University of Wisconsin Systems ‐ Milwaukee Milwaukee Wisconsin USA; ^8^ Themba Lethu Clinic Helen Joseph Hospital Johannesburg South Africa

**Keywords:** antiretroviral therapy, cognitive behavioural therapy, common elements treatment approach, intimate partner violence, mental and behavioural health, randomized controlled trial, retention in care, South Africa, sub‐Saharan Africa, viral load suppression

## Abstract

**Introduction:**

In South Africa, which has the world's highest prevalence of HIV, intimate partner violence (IPV) and common mental health conditions are barriers to retention in HIV treatment and achieving viral suppression. The Common Elements Treatment Approach (CETA), a cognitive‐behavioural‐therapy‐based intervention designed for lay healthcare worker delivery, is effective in reducing mental and behavioural health problems but has not been trialled for effectiveness in improving HIV outcomes. We conducted a randomized control trial to evaluate the effectiveness of CETA in improving HIV treatment outcomes among women experiencing IPV in South Africa.

**Methods:**

This was a single‐blind trial conducted among women living with HIV on antiretroviral therapy (ART) who experienced sexual and/or physical IPV in the last 12 months and had either an unsuppressed viral load or were at risk for poor medication adherence in the past year (e.g. defaulted on treatment, late or missed clinic visit). Women were randomized 1:1 to receive either 8–12 CETA counselling sessions or weekly safety text messages. The primary outcome was viral suppression (≤50 copies/mL) (which requires retention) at 12 months after baseline, which was evaluated using routinely collected medical records. A linear regression model was estimated to calculate risk differences and 95% confidence intervals (CI).

**Results:**

Participants were enrolled from 11 November 2021 to 19 July 2023, with 202 women randomized to CETA and 197 randomized to the control arm. Median age was 41.0 years (interquartile range [IQR]: 34.0, 47.0) with a median time on ART of 8.2 years (IQR: 4.3, 12.5). Receiving CETA was associated with a 1‐percentage point (95% CI: −0.11, 0.09) decrease in retention and viral suppression compared to the control. When restricted to individuals who completed CETA (*N* = 144) compared to all controls, we observed a 7‐percentage point (95% CI: −0.03, 0.18) increase in retention and viral suppression.

**Conclusions:**

Our estimates suggest overall there was no meaningful difference in retention and viral suppression at 12 months between individuals who received CETA compared to individuals who received the active safety control. Future work should seek to determine if CETA can be effective among those who complete the intervention.

## 1 Introduction

South Africa has the world's highest HIV prevalence, with almost eight million people living with HIV (PLWH) [1]. South Africa's National Antiretroviral Therapy programme has been instrumental in reducing morbidity and increasing life expectancy. As a result, tremendous progress has been made towards the UNAIDS 95‐95‐95 targets. In 2024, 95% of PLWH knew their status, 85% of those were on antiretroviral therapy (ART), and of those, 92% were virally suppressed [1]. While South Africa has achieved the first 95, there is more work to do on the latter two. Improving adherence and suppression is instrumental in preventing onward transmission and achieving UNAIDS targets by 2030 [2, 3].

Currently, technology (e.g. providing medication reminders [4]) and adherence counselling (e.g. educating patients on the importance of treatment adherence [5, 6]) have shown limited benefits. These interventions do not address some of the key barriers that individuals face across their lifetime on ART, including mental health‐related barriers. While some interventions have targeted improved mental health, effects have been mixed [7]. Most mental health interventions have targeted a single mental health condition, despite evidence that co‐occurring conditions, particularly depression, substance use, anxiety and trauma‐related disorders, are the norm among PLWH and are associated with poorer HIV treatment outcomes [8, 9, 10, 11, 12, 13, 14, 15, 16].

Intimate partner violence (IPV) is a major barrier to ART adherence and HIV care engagement, in part through its effects on mental health. It is estimated that about 35%–50% of South African women have experienced lifetime physical and/or sexual violence, most often by an intimate partner [17, 18]. Prior research has demonstrated IPV is associated with poorer retention in HIV care, lower ART adherence and reduced viral suppression [19]. Women experiencing IPV are also at increased risk for co‐occurring mental and behavioural health conditions, including depression, substance use and trauma‐related disorders [8], all of which are independently associated with poor HIV treatment outcomes. Given this, integrating IPV screening and support into HIV care programmes may improve ART adherence and broader outcomes among women living with HIV in South Africa [10, 20, 21].

The Common Elements Treatment Approach [22, 23, 24, 25, 26] (CETA) is an evidence‐based intervention addressing multiple problems related to substance use and mental health such as depression, trauma and violence. CETA is a transdiagnostic approach with flexibility in element implementation, allowing for treatment tailored to client needs. CETA has shown effectiveness at improving mental health and reducing IPV in clinical trials in Iraq [24], Thailand [25], Zambia [26, 27] and Colombia [28]. While a pilot study among Zambian adults living with HIV was conducted evaluating the effect of CETA on unhealthy alcohol use [27], CETA's effectiveness at improving HIV outcomes has not yet been evaluated.

Given CETA's prior success at improving mental health, substance use and IPV‐related outcomes and given that adherence to HIV care is also impacted by these conditions, CETA could improve adherence and retention in HIV care. We sought to test the effectiveness of CETA versus an active attention control on viral suppression and retention as well as mental health outcomes among women experiencing IPV who had issues with HIV care adherence. We separately published results of secondary outcomes on IPV and mental health outcomes [29], and here present results of our primary trial HIV outcomes.

## 2 Methods

We conducted a single‐blind, parallel‐arm, individually randomized controlled superiority trial at two outpatient HIV clinics in Johannesburg and Soweto, South Africa. One was a primary care clinic, while the other was a clinic at a secondary/tertiary care hospital. Both arms received reminders of clinic visits and had regular safety checks. Protocol details are presented elsewhere [30].

### 2.1 Eligibility

We initially enrolled adult (≥18 years old) virally unsuppressed (most recent viral load ≥50 copies/mL) HIV‐positive women on ART who experienced IPV in the past 12 months, could speak and read in English, Zulu or SeSotho and had their own cell phone for safety follow‐ups (though a cell phone could be provided). We excluded those unwilling to provide consent, currently psychotic or on unstable psychiatric regimens, had a suicide attempt or ideation with intent and plan and/or self‐harm in the past month, and those enrolled in another HIV intervention study. As initial enrolment was slow due to few eligible unsuppressed persons, we modified enrolment criteria from most recent viral load ≥50 copies/mL to be either most recent viral load ≥50 copies/mL, defaulted from treatment (i.e. missed or late [>14 days] visit in the last year).

Clinic staff unaffiliated with the study identified potentially eligible women during adherence counselling sessions and discussed the study. Those interested went through a brief consent process for eligibility screening which involved: (1) checking medical records for viral non‐suppression/missed visits; and (2) the physical/sexual violence subscale (27 items) of the Severity of Violence Against Women Scale [31] (SVAWS) via an audio computer‐assisted self‐interviewing (ACASI) tablet‐based questionnaire [32]. For those meeting all inclusion criteria, a full informed consent process was conducted, which included permission to access clinic records. Women were enrolled from 11 November 2021 to 19 July 2023, and the last date of data collection was 12 September 2024.

### 2.2 Randomization

Women were randomized 1:1 using blocked randomization (blocks of 4) through computer‐generated random number sequences created by MPF and linked to a REDCap database that revealed a new randomization arm linked to a study ID as each participant was enrolled to ensure randomization could not be manipulated.

### 2.3 Blinding

As the intervention used a specific therapy, we could not blind women and their caregivers, but the data analysis team was blinded to allocation until all 12‐month outcomes were collected.

### 2.4 Intervention

Participants randomized to the intervention arm received CETA, described in detail elsewhere [23, 27, 30, 33]. The main elements are provided in Figure S1. CETA uses elements of cognitive behavioural therapy (CBT) provided by trained lay providers to address symptoms of depression, traumatic stress, anxiety, substance misuse and other related problems (e.g. IPV, other safety issues, etc.). Providers received a 2‐week initial training and a manual on specific steps. Training followed an apprenticeship model [34]; after 10 days in‐person training, counsellors implement CETA with supervision from a local supervisor using CBT elements in varying combinations of elements, order and dose tailored to client symptomatology, including substance use, mental or behavioural health problems, violence and adherence to HIV treatment. Local supervisors were selected based on aptitude for CETA and received supervisor training and supported 5–6 CETA counsellors in weekly case review discussions. To ensure fidelity, counsellors provided supervisors with an objective, step‐by‐step report of each session and received a score from 1 to 5 by supervisors across several domains including adherence to intervention steps, quality of homework review, effectiveness of encouraging participation and engaging the participant, clarity in explaining session content and rationale, and overall skill in delivering the intervention component. Local supervisors met weekly with a CETA Trainer (KM and SS) reporting qualitative feedback and the scores of each counsellor's sessions with CETA participants to ensure fidelity. If any steps were missed, incomplete and/or delivered with low quality, counsellors repeated those components in the subsequent session.

CETA participants had 6–12 weekly 60‐min sessions, with the total number of sessions determined by the counsellor and supervisors after assessing symptom severity and progress. Women could receive CETA at a location of their choosing, including at the clinic and/or in the community. Women could choose to also invite a male partner to receive CETA separately [23], but as so few chose this option (*n* = 15), we do not report outcomes stratified by male partner enrolment. Additional details regarding the intervention can be found in the TIDieR statement [35] (Table S1).

### 2.5 COVID

COVID‐19 impacted study enrolment and participation in CETA due to restrictions in clinic access and transportation services. We amended our protocol to allow women to complete CETA via telehealth (e.g. T‐CETA). However, few chose this option (*n* = 23; 11.5%).

### 2.6 Control

As CETA is an attention‐based intervention, we sought to ensure that any benefits from CETA were not due to attention rather than CETA. Both arms received HIV text‐message appointment reminders to improve retention, which have shown some benefits [36, 37]. Messages did not include HIV‐specific information and could be provided in coded format to ensure confidentiality and safety.

### 2.7 Safety

Both arms followed clinic protocols for people experiencing IPV and severe mental health crises, including referral to support services and a pamphlet with community resources, 24/7 hotlines and emergency numbers. We implemented safety protocols in both arms, including evaluating severity of violence and other safety issues (e.g. suicidal intent) and implementing intensive safety planning. We conducted weekly safety checks by two‐way SMS or in person, asking: (1) “Are you thinking of killing yourself?”; (2) “Do you have a plan to kill yourself?”; (3) “Do you have the means to kill yourself?”; and (4) “Are you at risk of serious injury or death from interpersonal violence?”. Questions could be coded to ensure patient safety. Follow‐up by telephone, home visit and reaching out to support contacts were implemented if no response was received. Participants who responded affirmatively to any question were called by study staff to assess risk and complete safety planning. For high‐risk safety issues, household visits and/or referral to higher levels of care (e.g. psychiatrists, hospitals, safety centres, etc.) were included in safety planning. Those who conducted safety checks were not blinded to treatment arm.

### 2.8 Follow Up

Data were collected at baseline, and at 3, 12 and 24 months post‐baseline. Outside of CETA visits, appointment reminders and safety follow‐ups, study staff had no patient contact, and no effort was made to ensure retention given our primary outcome was retention‐based.

### 2.9 Outcomes

Our primary outcome was viral suppression. As this can only be assessed among those with a viral load, this becomes a combined outcome of retained and virally suppressed (≤50 copies/mL) ascertained from routine clinic data [38, 39] as to not influence retention [40, 41] at 12 months post randomization and up to 18 months post‐enrolment to allow for flexibility in timing of viral load testing and visit scheduling. All patients should have a scheduled visit within this time frame. We specified secondary outcomes of: (1) changes in IPV, post‐traumatic stress disorder, depression and substance use from baseline to 3 and 12 months; and (2) the cost‐effectiveness of CETA (reported separately [29]).

Adverse events were any untoward medical occurrence for participants while enrolled. Serious adverse events (SAEs) were those that could result in death, be life‐threatening, result in inpatient hospitalization and/or extension of pre‐existing hospitalization, or in persistent or significant disability. We reported any instances of increase in IPV and whether the participant believed it was study‐related.

### 2.10 Sample Size

We estimated 40% of control arm participants would reach our primary outcome [42] and a 20‐percentage point increase in the proportion suppressed/retained between arms would be clinically meaningful. With 80% power and a two‐sided α = 0.05, using a chi‐squared test for independent proportions, our sample size required was 91 per arm (182 total) [23], which we increased to 400 for assessment of secondary outcomes.

### 2.11 Data Analysis

The primary analysis was by intention‐to‐treat, where we estimated risk differences and risk ratios with corresponding 95% confidence intervals (CI) for our primary outcome. We conducted the following secondary analyses: (1) a per‐protocol analysis excluding individuals who did not complete CETA (post hoc analysis), and (2) a sub‐group analysis restricted to individuals in the top 50% (e.g. median split) of baseline symptoms (threatened IPV, physical/sexual IPV, depression and trauma) regardless of treatment arm. Two other post hoc analyses were conducted: (1) evaluating baseline characteristics associated with dropout at 3 and 12 months and (2) evaluating baseline characteristics associated with withdrawal from the study among CETA participants.

### 2.12 Sensitivity Analyses

To assess robustness of our estimates, we conducted the following not prespecified sensitivity analyses: (1) retention/viral suppression (viral load ≤400 copies/mL); (2) retention/viral suppression (viral load ≤ 1000 copies/mL); (3) utilized a ±3‐month window around 12 months; (4) separate 12‐month retention and suppression outcomes; (5) among individuals retained without a viral load documented, we conducted fully conditional specification multiple imputation for viral suppressions; and (6) restricted to individuals virally suppressed at enrolment.

### 2.13 Ethics

The trial was registered with clinicaltrials.gov (NCT04242992), and the South African National Clinal Trials Register (DOH‐27‐092021‐7303) and reviewed by the University of Witwatersrand Human Research Ethics Committee (Medical) (M200353), and the Boston University (H‐39746) and Johns Hopkins (12546) IRBs. Approval for anonymized data analysis was given by Columbia University as non‐human subjects research (AAAS9661). We empanelled a three‐member DSMB, who reviewed quarterly safety data, with immediate reporting of all SAEs or unanticipated problems. The DSMB was empowered to stop the study but did not.

## 3 Results

**FIGURE 1:**
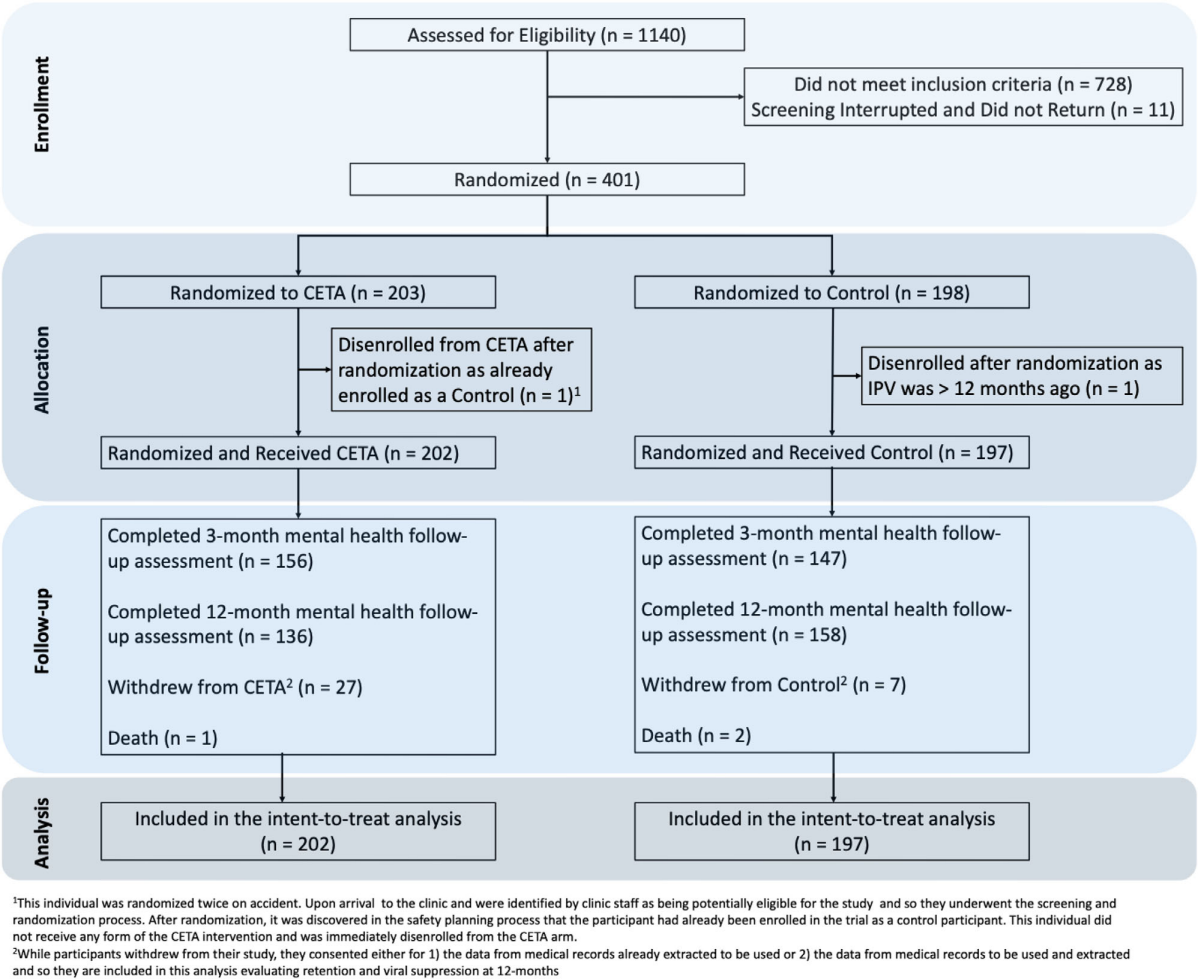
Study flow for individuals enrolled in a randomized trial of the effect of CETA on mental health outcomes in South Africa, among women with HIV on ART who have experienced violence and challenges with adherence. *CETA = Common Elements Treatment Approach.

Women were enrolled from 11 November 2021 to 19 July 2023. Of the 1140 who consented to screening, 401 were eligible for and enrolled into the study. Of the 401 individuals randomized, 203 were randomized to CETA and 198 to the control arm (Figure 1). Of the 203 randomized to CETA, one was disenrolled as they were already enrolled in the control arm. Of the 198 randomized to the control arm, one was disenrolled as the IPV they experienced was > 12 months prior, leaving 399 total participants.

The median age was 41.0 years (interquartile range [IQR]: 34.0, 47.0) with a median time on ART at 8.2 years (IQR: 4.3, 12.5) and a median viral load of 35 copies/mL (IQR: 20.0, 110.0; Table 1). Most were Black African (97.2%), never married (72.4%) and had missed a visit in the past year (60.4%). Baseline characteristics stratified by treatment arm were largely similar with both groups reporting high levels of depression (mean: 29.7, standard deviation [SD]: 12.1) and trauma (mean: 2.1, SD: 0.49). While all participants reported IPV in the past year, just over one‐third reported past 3‐month or ongoing IPV (31.5%; *n* = 126) and 36.6% (*n* = 146) were living with the IPV perpetrator. Of the 202 randomized to CETA, 144 (71.3%) completed CETA and averaged 7.6 sessions (SD: 5.5) over a mean of 12 weeks (SD: 7.0). Baseline mental health status (e.g. SVAWS, CES‐D and HTQ scores), relationship status, being late, missing and/or defaulting on treatment were not associated with withdrawal nor dropout at either timepoint (Tables S2 and S3). However, being virally suppressed at baseline was associated with withdrawal (odds ratio: 0.39, 95% CI: 0.16, 0.93) and dropout at 3 months (odds ratio: 0.62, 95% CI: 0.39, 0.99).

**TABLE 1:** Baseline demographics of individuals enrolled in a randomized trial of the effect of CETA on mental health outcomes in South Africa, among women with HIV on ART who have experienced violence and challenges with adherence by treatment arm (*N* = 399)^a^.

	CETA arm (*n* = 202)	Control arm (*n* = 197)	Total (*n* = 399)
**Age,** median (IQR)	40.0 [35.0, 46.0]	42.0 [34.0, 47.0]	41.0 [34.0, 47.0]
**Race**			
*Black African*	196 (97.0)	192 (97.5)	388 (97.2)
*Coloured*	2 (1.0)	5 (2.5)	7 (1.8)
*Missing*	4 (2.0)	0	4 (1.0)
**Education**			
*Grade 7 or less*	12 (6.0)	7 (3.6)	19 (4.8)
*Grades 8—10*	30 (14.9)	30 (15.2)	60 (15.0)
*Grades 11—12*	105 (52.0)	101 (51.3)	206 (51.6)
*Completed grade school*	34 (16.8)	45 (22.8)	79 (19.8)
*Certificate/diploma/post‐secondary*	12 (5.9)	13 (6.6)	25 (6.3)
*Degree*	4 (2.0)	1 (0.5)	5 (1.3)
*Unknown/missing*	5 (2.5)	0	5 (1.3)
**Currently in school**	14 (6.9)	17 (8.6)	31 (7.8)
**Employment**			
*Unemployed and looking for work*	141 (69.8)	120 (60.9)	261 (65.4)
*Unemployed and not looking for work*	5 (2.5)	3 (1.5)	8 (2.0)
*Self‐employed (part‐ or full‐time)*	12 (6.0)	21 (10.7)	33 (8.3)
*Employed part‐time*	23 (11.4)	29 (14.7)	52 (13.0)
*Employed full‐time*	15 (7.4)	21 (10.7)	36 (9.0)
*Other/missing*	6 (3.0)	3 (1.5)	9 (2.3)
**Currently living with perpetrator of IPV**	75 (37.1)	71 (36.0)	146 (36.6)
**Marital status**			
*Never married*	147 (72.8)	142 (72.1)	289 (72.4)
*Married*	35 (17.3)	30 (15.2)	65 (16.3)
*Divorced/separated*	13 (6.5)	14 (7.2)	27 (6.8)
*Widowed*	4 (2.0)	11 (5.6)	15 (3.8)
*Unknown/missing*	3 (1.5)	0	3 (0.8)
**Have a current partner**	178 (88.1)	181 (91.9)	359 (90.0)
**Have children**	176 (87.1)	172 (87.3)	348 (87.7)
**Suicidal ideation**	92 (45.5)	73 (37.1)	165 (41.4)
**Homicidal ideation**	47 (23.3)	43 (21.9)	90 (22.3)
**SVAWS Threatened Score**, mean (SD) [range]	38.9 (13.7) [19−75]	37.6 (12.4) [16−70]	38.2 (13.1) [16−75]
**SVAWS Physical Score**, mean (SD) [range]	50.9 (19.8) [28−108]	49.4 (17.3) [25−100]	50.2 (18.6) [25−108]
**CES‐D Score**, mean (SD) [range]	30.9 (12.2) [4−54]	28.5 (12.0) [1−55]	29.7 (12.1) [1−55]
**HTQ Score**, mean (SD) [range]	2.1 (0.5) [1.0−3.2]	2.0 (0.5) [1.0−3.0]	2.1 (0.49) [1.0−3.2]
**Missed a visit within the past year** ^b^	122 (60.4)	119 (60.4)	241 (60.4)
**Late to a visit within the past** year^b^	69 (34.2)	54 (27.4)	123 (30.8)
**Defaulted on treatment within the past** year^b^	114 (56.4)	113 (57.4)	227 (56.9)
**Years on ART**, median (IQR)	8.5 [4.8, 12.6]	8.0 [3.6, 12.5]	8.2 [4.3, 12.5]
**Viral load,** copies/mL, median (IQR)	30 (20, 107.5)	39 (20, 139.5)	35 (20, 110)
*0–50*	125 (61.9)	122 (61.9)	247 (61.9)
*51–500*	44 (21.8)	38 (19.3)	82 (20.6)
*501–1000*	5 (2.5)	3 (1.5)	8 (2.0)
*1001–5000*	8 (4.0)	7 (3.6)	15 (3.8)
*5001–50,000*	6 (3.0)	8 (4.1)	14 (3.5)
*50,001–500,000*	2 (1.0)	9 (4.6)	11 (2.8)
*500,001+*	2 (1.0)	1 (0.5)	3 (0.8)
*Missing*	10 (5.0)	9 (4.6)	19 (4.8)

Abbreviations: CES‐D = Center for Epidemiologic‐Studies Depression Scale (Possible Range: 0–60); CETA = Common Elements Treatment Approach; Control arm received 12 weeks of weekly SMS messaging; HTQ = Harvard Trauma Questionnaire (Possible Range: 1–4); SVAWS = Severity of Violence Against Women Scale (Threatened Scale Possible Range: 19–76; Physical/Sexual IPV Scale Possible Range: 27–108).

^a^Total *N* = 399 instead of 400 because there were individuals who were randomized and then disenrolled after randomization. See Figure 1 for more details.

^b^May sum to greater than the total sample size across these three markers of engagement with care as these are non‐mutually exclusive categories.

By the end of follow‐up, 34 participants (*n* = 27 CETA; *n* = 7 control) withdrew from the study but did not withdraw consent to use their data and three (*n* = 1 CETA; *n* = 2 control) deaths unrelated to study participation occurred (Figure 1).

### 3.1 Primary Analysis

At 12 months, of the 202 randomized to CETA, 86 (42.6%) were retained and virally suppressed, and 116 (57.4%) had a negative outcome (e.g. not retained, retained and no viral load documented, or retained and virally unsuppressed; Table 2). Of the 197 randomized to the control arm, at 12 months, 86 (43.7%) were retained in care and virally suppressed, and 111 (56.3%) had a negative outcome. This translated to no difference in retention and viral suppression (RD: 0.01; 95% CI: −0.11, 0.09; RR: 0.98; 95% CI: 0.78, 1.22; Table 2).

**TABLE 2:** Risk difference, risk ratio and 95% confidence intervals (CI) for retention and viral suppression of individuals enrolled in a randomized trial of the effect of CETA on mental health outcomes in South Africa, among women with HIV on antiretroviral therapy who have experienced violence and challenges with adherence using a 6‐month window.

	CETA arm	Control arm	Risk difference *(95% CI)*	Risk ratio *(95% CI)*
**Negative outcome at 12** months^a^			−0.01 (−0.11, 0.09)	0.98 (0.78, 1.22)
*Not retained*	49 (24.3)	38 (19.3)		
*Retained and no viral load documented*	36 (17.8)	43 (21.8)		
*Retained and unsuppressed*	31 (15.4)	30 (15.2)		
**Retained and suppressed at 12** months^a^ (primary outcome)	86 (42.6)	86 (43.7)		

Abbreviation: CETA; Common Elements Treatment Approach; Control arm received 12 weeks of weekly short message service (SMS) messaging.

^a^Virally suppressed was defined as a viral load ≤ 50 copies/mL. All other outcomes (e.g. not retained, retained and unsuppressed, retained but no viral load documented) were considered a negative outcome.

### 3.2 Secondary Analyses

Table 3 presents the effect of CETA on retention and suppression versus the control group restricted to: (1) participants who completed CETA; and (2) restricted to participants in the top 50% of baseline mental health symptoms (e.g. threatened IPV, physical and/or sexual IPV, depression and trauma) regardless of treatment arm. Excluding those who did not complete CETA (*n* = 56), we observed a small increase in 12‐month retention and suppression associated with CETA (RD: 0.07; 95% CI: −0.03, 0.18, RR: 1.18; 95% CI: 0.94, 1.47). Similarly, when restricted to individuals in the top 50% of symptoms for (1) threatened IPV, (2) physical and/or sexual IPV and (3) trauma effect, estimates were similar to our primary analysis. For the 198 individuals in the top 50% of threatened IPV, we observed a small, imprecise decrease in retention and viral suppression (RD: 0.06; 95% CI: −0.20, 0.08, RR: 0.86; 95% CI: 0.62, 1.20; Table 3). Limited to the 201 in the top 50% of physical/sexual IPV, we observed a small, imprecise decrease in retention and viral suppression (RD: −0.07; 95% CI: −0.21, 0.07, RR: 0.86, 95% CI: 0.63, 1.16). Restricted to the 202 in the top 50% of trauma symptoms (HTQ), we saw no difference in retention and suppression (RD: 0.0001; 95% CI: −0.14, 0.14, RR: 1.00; 95% CI: 0.74, 1.36). However, when restricted to the 201 in the top 50% of depressive symptoms (CE‐SD), we found a small, imprecise increase in retention and suppression (RD: 0.04; 95% CI: −0.10, 0.17, RR: 1.08; 95% CI: 0.81, 1.43).

**TABLE 3:** Risk difference, risk ratio and 95% confidence intervals for retention and viral suppression at 12 months of individuals enrolled in a randomized trial of the effect of CETA on mental health outcomes in South Africa, among women with HIV on antiretroviral therapy who have experienced violence and challenges with adherence using a 6‐month window when restricted to (1) individuals who complete CETA and (2) individuals in the top 50% of baseline mental health symptoms.

	CETA arm	Control arm	Risk difference *(95% confidence interval)*	Risk ratio *(95% confidence interval)*
**Completed CETA** (*n* = 343)				
Retained and suppressed^a^	74 (51.4)	86 (43.7)	0.07 (−0.03, 0.18)	1.18 (0.94, 1.47)
**SVAWS Threatening Violence** (CETA arm = 107; Control arm *n* = 97)				
Retained and suppressed^a^	41 (38.3)	43 (44.3)	−0.06 (−0.20, 0.08)	0.86 (0.62, 1.20)
**SVAWS Physical and Sexual Violence** (CETA arm = 103; Control arm *n* = 98)				
Retained and suppressed^a^	43 (45.3)	48 (50.0)	−0.07 (−0.21, 0.07)	0.86 (0.63, 1.16)
**HTQ** (CETA arm = 111; Control arm *n* = 91)				
Retained and suppressed^a^	50 (45.1)	41 (45.1)	−0.0001 (−0.14, 0.14)	1.00 (0.74, 1.36)
**CES‐D** (CETA arm = 103; Control arm *n* = 98)				
Retained and suppressed^a^	58 (50.4)	43 (46.7)	0.04 (−0.10, 0.17)	1.08 (0.81, 1.43)

Abbreviations: CES‐D = Center for Epidemiologic‐Studies Depression Scale (Possible Range: 0–60); CETA = Common Elements Treatment Approach; Control arm received 12 weeks of weekly SMS messaging; HTQ = Harvard Trauma Questionnaire (Possible Range: 1–4); SVAWS = Severity of Violence Against Women Scale (Threatening Violence Scale Possible Range: 19–76; Physical/Sexual Violence Scale Possible Range: 27–108).

^a^Virally suppressed was defined as a viral load ≤ 50 copies/mL. All other outcomes (e.g. not retained, retained and unsuppressed, retained but no viral load documented) were considered a negative outcome.

### 3.3 Sensitivity Analyses

In our six sensitivity analyses to evaluate the robustness of our estimates, all were consistent with no difference in 12‐month retention and viral suppression (Tables S4–S7).

### 3.4 Adverse Events

We observed 26 adverse events in the CETA arm, of which 12 were SAEs and five in the control arm, of which three were SAEs. We had far more contact with those in the CETA arm, so this may reflect reporting bias due to increased contact. No SAE was related to the study. There were 10 post‐baseline reports of increased violence, all related to an adverse event. Seven were evaluated as unrelated to study participation, two were unlikely to be related and only one was possibly related.

## 4 Discussion

While this is the fourth CETA clinical trial in an low and middle income country (LMIC) PTS among violence‐affected populations, this is the first where the primary outcome was HIV retention/viral suppression. Our findings suggest no meaningful change in retention or suppression at 12 months for those who received CETA versus the active control. In this violence‐affected population of women at risk for poor adherence, retention and suppression rates were low overall, but with no important impact of CETA. These results are in line with systematic reviews that found interventions such as CBT, adherence reminder devices and education programmes aimed at improving suppression and retention in care have had limited impact on HIV treatment outcomes [6, 43, 44, 45, 46, 47]. This is in contrast with CETA's documented effect on mental health outcomes [24, 25, 27, 28, 48] and substance abuse [26, 27], where trials have shown clear benefits for both. The current study found clear, sustained improvement for mental health conditions, including depression and trauma [29].

There are three potential reasons CETA had little effect on retention and viral suppression. First, this may be due to high ART effectiveness, particularly dolutegravir. Dolutegravir, which most of our participants were on, is highly effective in helping individuals achieve and remain suppressed [49, 50, 51]. Furthermore, as we expanded our eligibility criteria to include those suppressed but with missed visits, 62% were suppressed at baseline. Most were eligible due to either being late for or missing a visit in the past year, which does not inherently indicate adherence issues. However, when restricted to those completing CETA, we found a small, imprecise increase in retention/suppression (RD: 0.07, 95% CI: −0.03, 0.18). Second, we hypothesized retention and suppression would be improved by reducing IPV. However, as CETA did not reduce IPV in this population, this could explain why CETA had little effect on retention and suppression [29]. Third, we used an active control arm with weekly safety checks as we could not ethically design a study with a true control. However, as a result, the active control arm may have reduced IPV symptoms and co‐occurring mental health problems, potentially contributing to our null findings.

Future studies should explore whether symptom severity impacts CETA effectiveness on HIV outcomes. While we observed an effect on viral suppression at 3 months, it was attenuated by 12 months in our sensitivity analysis, and few had a documented 3‐month viral load.

Retention has proven difficult to impact. Despite strong improvements in retention and suppression over the last decade [52, 53, 54], retention rates globally remain suboptimal. Our recent review estimated retention in LMICs was between 67% and 75% at 36 months [55]. Recent focus on differentiated HIV care seeks to tackle this problem, but for those experiencing poor adherence and loss to follow‐up, these interventions have had limited impact [56, 57]. Our own evaluation of South Africa's National Adherence Guidelines for Chronic Diseases using differentiated care found benefits for those already adhering to care schedules and virally suppressed [58, 59], but not for patients not retained and with poor adherence [42]. This suggests even targeted interventions like CETA may not be able to overcome systemic barriers that prevent high adherence and retention. Systemwide interventions may be necessary [29].

Our study has several limitations. First, use of a 6‐month window to account for flexibility in timing and scheduling of clinic and viral load appointments may not precisely reflect a 12‐month measurement. Second, individuals at one of the clinics are typically more complex HIV cases and often travel from outside of Johannesburg to receive care, while the other is more representative of the local community. Third, as our study population is at high‐risk for self‐harm and violence (suicide, homicide and abuse), we were unable to ethically design a study with a true control. As such, it is possible safety checks provided to controls reduced IPV, depressive and trauma symptoms and could have improved retention and suppression [60]. Next, as we had to change our eligibility criteria to include those at high risk of loss to follow‐up but not unsuppressed, this could have reduced our power to detect effects. Still, given they were at high risk of being lost and our primary outcome inherently includes loss, we do not think this was a major driver of our mostly null results. In addition, visits can occur and not be documented in clinic files or get documented in a way that makes it difficult to see them in our dataset (e.g. incorrect dates listed). As such, our retention measure could have some misclassification, but this is likely non‐differential with respect to the intervention. Finally, we did not have a pure control arm as SMSing can change retention in HIV care. However, we feel this is still an appropriate control as an active attention control is necessary to see if there is any effect of CETA directly and not just providing any attention.

## 5 Conclusions

Results from our study suggest that CETA had little to no effect on retention in care and viral suppression at 12 months compared to the active control arm. Although CETA did not improve HIV treatment outcomes, its proven benefits for depression and post traumatic stress disorder highlight its potential role as an essential mental health intervention in HIV care programmes, particularly in settings where mental health services are limited. Therefore, CETA is still beneficial, especially as mental healthcare services are highly limited in South Africa.

## Author Contributions

Conceived of and designed the work: MPF, LKM, DT, JCK, SP and LL. Contributed to designing the work: MPF, LKM, DMT, JCK, SP, KM, SS, SM‐N, TA, LL, BG and MV. Wrote the first draft of the manuscript: MPF and AZ. Acquisition of data: RG, AZ, SM‐N, PM and SS. Data analysis: AZ. Reviewed and revised the manuscript: All authors. Approved the manuscript's results and conclusions: All authors. All authors have read, and confirm that they meet, ICMJE criteria for authorship.

## Funding

Research reported in this publication is supported by the National Institute of Mental Health of the National Institutes of Health under Award Number R01MH121998. LL is supported by the National Institute of Mental Health of the National Institutes of Health under grant number K01MH119923. JCK's contribution is supported in part by the National Institute on Alcohol Abuse and Alcoholism (NIAAA) under grant number K01AA026523. AZ is supported in part by the National Institute on Drug Abuse under grant number 5T32DA013911. The funders had no role in study design, data collection and analysis, decision to publish or preparation of the manuscript.

## Disclaimer

The content is solely the responsibility of the authors and does not necessarily represent the official views of the National Institutes of Health.

## Ethics Approval and Consent to Participate

The study protocol has been approved by the Boston University Institutional Review Board (H‐39746), the University of the Witwatersrand Human Research Ethics Committee (Medical) (M200353) and the Johns Hopkins Institutional Review Board (12546). The study was approved as non‐human subjects by the Columbia University Institutional Review Board (AAAS9661). Written informed consent was obtained from all study participants prior to enrolment by study staff.

## Conflicts of Interest

The authors declare that they have no conflicts of interest.

## Supporting information




**Table S1**: TIDieR statement for reporting of interventions [35]
**Table S2**: Baseline characteristics and demographics at enrolment and their association with loss to follow‐up at 3 and 12 months of follow‐up
**Table S3**: Baseline characteristics and demographics at enrolment and their association with withdrawal from the study among CETA participants
**Table S4**: Sensitivity analysis 1, 2 and 3: risk difference, risk ratio and 95% confidence intervals for retained and virally suppressed at 12 months of individuals enrolled in a randomized trial of the effect of CETA on mental health outcomes in South Africa, among women with HIV on ART who have experienced violence and challenges with adherence [1]
**Table S5**: Sensitivity analysis 4: risk difference, risk ratio and 95% confidence intervals for retention and viral suppression at 12 months of individuals enrolled in a randomized trial of the effect of CETA on mental health outcomes in South Africa, among women with HIV on ART who have experienced violence and challenges with adherence using a 6‐month window
**Table S6**: Sensitivity analysis 5: risk difference, risk ratio and 95% confidence intervals for retention and viral suppression at 12 months of individuals enrolled in a randomized trial of the effect of CETA on mental health outcomes in South Africa, among women with HIV on ART who have experienced violence and challenges with adherence using 20 multiply imputed datasets [1]
**Table S7**: Sensitivity analysis 6: risk difference, risk ratio and 95% confidence intervals for retention and viral suppression at 12 months of individuals enrolled in a randomized trial of the effect of CETA on mental health outcomes in South Africa, among women with HIV on ART who have experienced violence and challenges with adherence restricted to individuals who were virally suppressed at enrolment (*N* = 247) [1]
Figure S1—CETA main elements

## Data Availability

Data generated by the study will be made available in de‐identified format following protocol closure in a publicly available repository, to be identified in papers published from this study. Data obtained from the study sites (routinely generated medical record data) will not be owned by the authors and cannot be made available by them.
