# Supplementary material for: The Impact of the Common Elements Treatment Approach on HIV Treatment Outcomes Among Women Experiencing Intimate Partner Violence in South Africa: A Randomized Trial
Source: J Int AIDS Soc. 2026 Jul 27;29(8):e70172. doi: 10.1002/jia2.70172 (PMC13402931; doi:10.1002/jia2.70172)

**Supplemental Table 1.** TIDieR Statement for Reporting of Interventions^35^

**Supplemental Table 2.** Baseline characteristics and demographics at enrollment and their association with loss to follow-up at 3- and 12-months of follow-up

|  | **Odds Ratio**  **(95% Confidence Interval)** | |
| --- | --- | --- |
|  | *3-months* | *12-months* |
| **Age** | 0.97 (0.95, 1.00) | 0.96 (0.93, 0.98) |
| **Race** |  |  |
| *Coloured* | 1.27 (0.24, 6.65) | 7.01 (1.34, 36.69) |
| *Black African* | 1.00 (Ref) | 1.00 (Ref) |
| **Attended School** | 0.95 (0.20, 4.78) | 1.00 (1.00, 1.00) |
| **Currently Enrolled in School** | 1.32 (0.58, 2.97) | 1.11 (0.49, 2.49) |
| **Employed** | 2.46 (0.91, 2.36) | 0.89 (0.55, 1.44) |
| **Married** | 1.11 (0.66, 1.86) | 1.39 (0.83, 2.33) |
| **Have a Partner** | 1.16 (0.51, 2.63) | 1.18 (0.54, 2.60) |
| **Cohabitation Status** |  |  |
| *With husband* | 0.95 (0.48, 1.88) | 0.86 (0.52, 1.43) |
| *With regular partner* | 0.92 (0.47, 1.82) | 1.29 (0.80, 2.08) |
| *Not cohabitating with anyone* | 1.00 (Ref) | 1.00 (Ref) |
| **Defaulted on Treatment** | 0.91 (0.58, 1.45) | 1.00 (0.63, 1.56) |
| **Late to a Visit** | 1.04 (0.63, 1.73) | 1.26 (0.75, 2.07) |
| **Missed a Visit** | 1.21 (0.74, 1.98) | 1.05 (0.65, 1.71) |
| **Virally Suppressed**^1^ | 0.62 (0.39, 0.99) | 0.83 (0.53, 1.30) |
| **Baseline HTQ Score**^2^ | 0.73 (0.53, 1.00) | 1.00 (0.74, 1.35) |
| **Baseline CES-D Score**^2^ | 0.99 (0.97, 1.01) | 0.99 (0.97, 1.01) |
| **Baseline SVAWS Threatened Score**^2^ | 0.99 (0.97, 1.01) | 1.00 (0.99, 1.02) |
| **Baseline SVAWS Physical Score**^2^ | 1.00 (0.99, 1.01) | 1.00 (0.99, 1.02) |

^1^Virally suppressed at baseline was defined as a viral load < 50 copies/mL

^2^SVAWS = Severity of Violence Against Women Scale (Threatened Scale Possible Range: 19-76; Physical/Sexual IPV Scale Possible Range: 27-108), CES-D = Center for Epidemiologic-Studies Depression Scale (Possible Range: 0-60); HTQ = Harvard Trauma Questionnaire (Possible Range: 1-4).

**Supplemental Table 3.** Baseline characteristics and demographics at enrollment and their association with withdrawal from the study among CETA participants

|  | **Odds Ratio**  *(95% Confidence Interval)* |
| --- | --- |
| **Age** | 0.93 (0.87, 0.98) |
| **Race** |  |
| *Coloured* | 8.33 (0.50, 138.21) |
| *Black African* | 1.00 (Ref) |
| **Currently Enrolled in School** | 2.35 (0.60, 9.19) |
| **Employed** | 2.546 (1.22, 4.94) |
| **Married** | 1.14 (0.43, 3.04) |
| **Have a Partner** | 0.76 (0.21, 2.81) |
| **Cohabitation Status** | 0.93 (0.72, 1.20) |
| *With husband* | 0.85 (0.22, 3.25) |
| *With regular partner* | 1.17 (0.45, 3.00) |
| *Not cohabitating with anyone* | 1.00 (Ref) |
| **Defaulted on Treatment** | 0.90 (0.38, 2.12) |
| **Late to a Visit** | 0.63 (0.23, 1.72) |
| **Missed a Visit** | 0.76 (0.26, 2.27) |
| **Virally Suppressed**^1^ | 0.39 (0.16, 0.93) |
| **Baseline HTQ Score**^2^ | 0.56 (0.31, 1.01) |
| **Baseline CES-D Score**^2^ | 0.95 (0.92, 1.00) |
| **Baseline SVAWS Threatened Score**^2^ | 0.99 (0.95, 1.02) |
| **Baseline SVAWS Physical Score**^2^ | 0.99 (0.98, 1.01) |

^1^Virally suppressed at baseline was defined as a viral load < 50 copies/mL

^2^SVAWS = Severity of Violence Against Women Scale (Threatened Scale Possible Range: 19-76; Physical/Sexual IPV Scale Possible Range: 27-108), CES-D = Center for Epidemiologic-Studies Depression Scale (Possible Range: 0-60); HTQ = Harvard Trauma Questionnaire (Possible Range: 1-4).

**Supplemental Table 4.** Sensitivity Analysis 1, 2, and 3: Risk Difference, Risk Ratio, and 95% Confidence Intervals for Retained and Virally Suppressed at 12-months of individuals enrolled in a randomized trial of the effect of CETA on mental health outcomes in Johannesburg South Africa among women with HIV on ART who have experienced violence and challenges with adherence^1^

|  | **Risk Difference**  *(95% Confidence Interval)* | **Risk Ratio**  *(95% Confidence Interval)* |
| --- | --- | --- |
| **Retained and Virally Suppressed**^2^ | -0.03 (-0.13, 0.07) | 0.95 (0.78, 1.14) |
| **Retained and Virally Suppressed**^3^ | -0.003 (-0.10, 0.09) | 0.99 (0.83, 1.20) |
| **Retained and Virally Suppressed**^4^ | -0.02 (-0.11, 0.07) | 0.92 (0.68, 1.26) |

^1^All other outcomes (e.g., not retained, retained and unsuppressed, retained but no viral load documented) were considered a negative outcome

^2^Virally suppressed was defined as a viral load < 400 copies/mL, a 6-month window was used

^3^Virally suppressed was defined as viral load < 1000 copies/mL, a 6-month window was used

^4^Virally suppressed was defined as viral load < 50 copies/mL, a 3-month window was used

**Supplemental Table 5.** Sensitivity Analysis 4: Risk Difference, Risk Ratio, and 95% Confidence Intervals for Retention and Viral Suppression at 12-months of individuals enrolled in a randomized trial of the effect of CETA on mental health outcomes in Johannesburg South Africa among women with HIV on ART who have experienced violence and challenges with adherence using a 6-month window

|  | **CETA Arm** | **Control Arm** | **Risk Difference**  *(95% Confidence Interval)* | **Risk Ratio**  *(95% Confidence Interval)* |
| --- | --- | --- | --- | --- |
| **Retained in Care**^1^ | 153 (75.7) | 159 (80.7) | -0.05 (-0.13, 0.03) | 0.94 (0.85, 1.04) |
| **Virally Suppressed**^2^ | 86 (50.0) | 32 (51.6) | -0.01 (-0.13, 0.10) | 0.98 (0.84, 1.15) |

^1^Retained in care was defined as an individual attending a visit at the clinic 12-months from enrollment with a +6-month window, regardless of viral suppression status

^2^Among individuals with a documented viral load, virally suppressed was defined as viral load < 50 copies/mL, a 6-month window was used

**›**

**Supplemental Table 6.** Sensitivity Analysis 5: Risk Difference, Risk Ratio, and 95% Confidence Intervals for Retention and Viral Suppression at 12-months of individuals enrolled in a randomized trial of the effect of CETA on mental health outcomes in Johannesburg South Africa among women with HIV on ART who have experienced violence and challenges with adherence using 20 multiply imputed datasets^1^

|  | **Risk Difference**  *(95% Confidence Interval)* | **Risk Ratio**  *(95% Confidence Interval)* |
| --- | --- | --- |
| **Retained and Suppressed**^2^ | -0.03 (-0.14, 0.08) | 0.96 (0.83, 1.11) |

^1^ In addition to the outcome, the following covariates were included in the imputation: treatment arm, site, financial hardship, employment status, tobacco use, alcohol use, education level, marital status, age at baseline, time on ART, and viral load at baseline. After removal of 400 iterations in the burn-in period, 20 imputed datasets were created with SAS PROC MI and combined coefficient and standard error estimates from the datasets using Rubin’s Rules. Convergence was assessed via visual inspection of the individual parameter trace plots which indicated no convergence issues.

^2^Virally suppressed was defined as a viral load < 50 copies/mL; All other outcomes (e.g., not retained, retained and unsuppressed, retained but no viral load documented) were considered a negative outcome

**Supplemental Table 7.** Sensitivity Analysis 6: Risk Difference, Risk Ratio, and 95% Confidence Intervals for Retention and Viral Suppression at 12-months of individuals enrolled in a randomized trial of the effect of CETA on mental health outcomes in Johannesburg South Africa among women with HIV on ART who have experienced violence and challenges with adherence restricted to individuals who were virally suppressed at enrollment (N = 247)^1^

|  | **Risk Difference**  *(95% Confidence Interval)* | **Risk Ratio**  *(95% Confidence Interval)* |
| --- | --- | --- |
| **Retained and Suppressed**^2^ | 0.04 (-0.09, 0.16) | 1.09 (0.82, 1.44) |

^2^Virally suppressed was defined as a viral load < 50 copies/mL; All other outcomes (e.g., not retained, retained and unsuppressed, retained but no viral load documented) were considered a negative outcome

Supplemental Figure 1 - CETA main elements
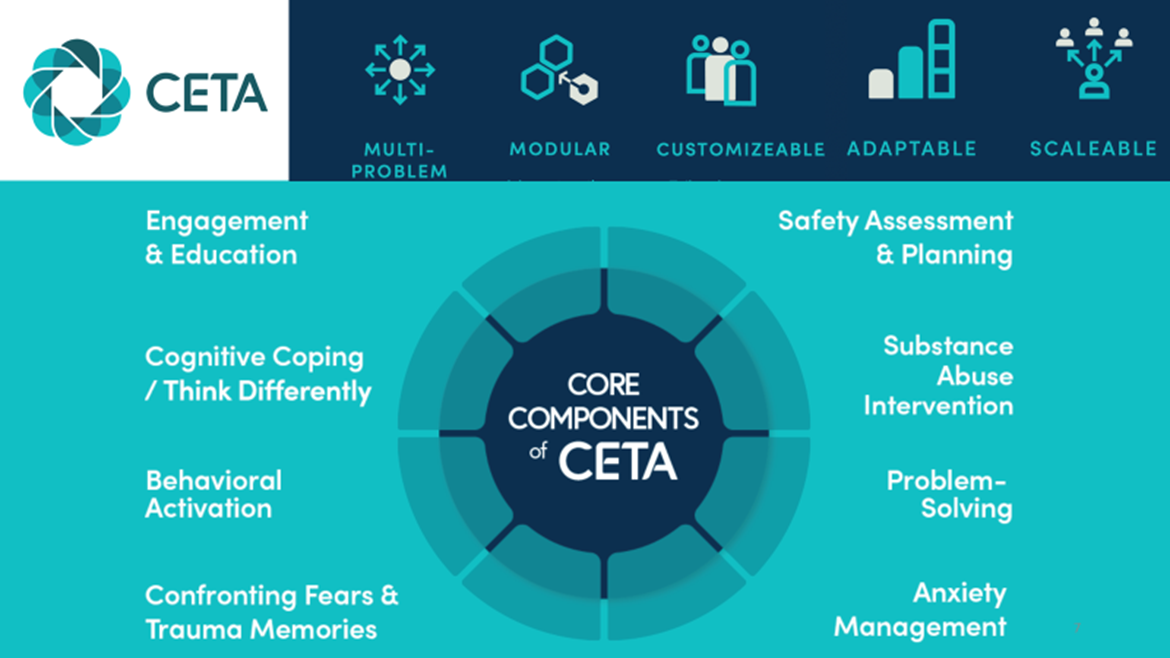

Supplement: Supplementary file 1 — Table S1: TIDieR statement for reporting of interventions [35] Table S2: Baseline characteristics and demographics at enrolment and their association with loss to follow‐up at 3 and 12 months of follow‐up Table S3: Baseline characteristics and demographics at enrolment and their association with withdrawal from the study among CETA participants Table S4: Sensitivity analysis 1, 2 and 3: risk difference, risk ratio and 95% confidence intervals for retained and virally suppressed at 12 months of individuals enrolled in a randomized trial of the effect of CETA on mental health outcomes in South Africa, among women with HIV on ART who have experienced violence and challenges with adherence [1] Table S5: Sensitivity analysis 4: risk difference, risk ratio and 95% confidence intervals for retention and viral suppression at 12 months of individuals enrolled in a randomized trial of the effect of CETA on mental health outcomes in South Africa, among women with HIV on ART who have experienced violence and challenges with adherence using a 6‐month window Table S6: Sensitivity analysis 5: risk difference, risk ratio and 95% confidence intervals for retention and viral suppression at 12 months of individuals enrolled in a randomized trial of the effect of CETA on mental health outcomes in South Africa, among women with HIV on ART who have experienced violence and challenges with adherence using 20 multiply imputed datasets [1] Table S7: Sensitivity analysis 6: risk difference, risk ratio and 95% confidence intervals for retention and viral suppression at 12 months of individuals enrolled in a randomized trial of the effect of CETA on mental health outcomes in South Africa, among women with HIV on ART who have experienced violence and challenges with adherence restricted to individuals who were virally suppressed at enrolment (N = 247) [1] Figure S1—CETA main elements [file JIA2-29-e70172-s001.docx]
